# Perception of Faces and Elaboration of Gender and Victim/Aggressor Stereotypes: The Influence of Internet Use and of the Perceiver’s Personality

**DOI:** 10.3389/fpsyg.2021.561480

**Published:** 2021-06-22

**Authors:** Margherita Bracci, Stefano Guidi, Enrica Marchigiani, Maurizio Masini, Paola Palmitesta, Oronzo Parlangeli

**Affiliations:** Department of Social, Political and Cognitive Sciences, Dispoc, University of Siena, Siena, Italy

**Keywords:** gender, social media, past events of aggression, cybervictimization, personality, empathy

## Abstract

The use of social media, particularly among youngsters, is characterized by simple and fast image exploration, mostly of people, particularly faces. The study presented here was conducted in order to investigate stereotypical judgments about men and women concerning past events of aggression—perpetrated or suffered—expressed on the basis of their faces, and gender-related differences in the judgments. To this aim, 185 participants answered a structured questionnaire online. The questionnaire contained 30 photos of young people’s faces, 15 men and 15 women (Ma et al., 2015), selected on the basis of the neutrality of their expression, and participants were asked to rate each face with respect to masculinity/femininity, strength/weakness, and having a past of aggression, as a victim or as a perpetrator. Information about the empathic abilities and personality traits of participants were also collected. The results indicate that the stereotypes—both of gender and those of victims and perpetrators—emerge as a consequence of the visual exploration of faces that present no facial emotion. Some characteristics of the personality of the observers, such as neuroticism, extraversion, openness, conscientiousness, and affective empathy, have a role in facilitating or hindering stereotype processing, in different ways for male and female faces by male and female observers. In particular, both genders attribute their positive stereotypical attributes to same-gender faces: men see male faces as stronger, masculine, and more aggressive than women do, and women see female faces as more feminine, less weak, and less as victims than men do. Intensive use of social media emerges as a factor that could facilitate the expression of some stereotypes of violent experiences and considering female subjects as more aggressive. Findings in this study can contribute to research on aggressive behavior on the Internet and improve our understanding of the multiple factors involved in the elaboration of gender stereotypes relative to violent or victim behavior.

## Introduction

The use of the Internet and social networking sites (SNSs) has greatly increased in recent years. Today about half of the world’s population, 3.8 billion people, regularly use social networks, an increase of about 9% compared to 2019, and they spend, on average, 2 h and 24 min per day on social media (Report Digital, 2020).

In Italy, 35 million of almost 50 million people online are active on a social network. According to a study by the Italian National Adolescence Monitoring Agency—(Osservatorio Nazionale Adolescenza, 2017), despite the fact that for people under 13 years of age opening an account on social media is not allowed by law, almost 78% of the youth (in a sample of 3,900) from 11 to 13 years old have a social profile. Nearly 15% of the observed sample group spends 10 h on their smartphone a day, 18% from 7 to 10 h, and the majority of these are girls. The reasons for this exponential expansion of SNSs among youngsters are numerous, though the relational aspect seems to be dominant. For many users the most attractive aspect of social media is the possibility of looking through many different user profiles, and through this, establishing new personal relationships (Muscanell and Guadagno, 2012). To this aim, and in this process, it is clear that the representations of human faces in images play a fundamental role. According to a recent study, the photos of faces posted on social networks have 38% more chance of getting a “like,” and 32% more chance of receiving a comment compared to other kinds of posted photos, and this independently from the sex and age of the subject of the photograph. Online communication, in fact, is characterized by the pervasive presence of photos of people, mostly faces (Bakhshi et al., 2014).

Faces in social media, as in actual relationships, often represent the first and only clue on which we base our first impression of someone we do not know (Willis and Todorov, 2006). The perception of the characteristics of a face can even influence the first impression of an individual regarding people’s personality traits. Numerous studies show how people are able to make judgments about an individual’s personality after only a few milliseconds of exposure to an “emotionally neutral” face (Bar et al., 2006; Willis and Todorov, 2006). For example, individuals with infantile facial characteristics (round face, big eyes, high eyebrows, and small nose) are perceived as more naïve, more affectionate, less dominant, and inspire more trust—the baby-face overgeneralization effect (Oosterhof and Todorov, 2009; Todorov et al., 2011). However, a face can also suggest a certain vulnerability or weakness; in this case a person can be perceived as a possible victim of a virtual aggression (Ciucci et al., 2014). The face, further, is one of the very first stimuli individuals use as a cue to establish the propensity of aggressiveness of another individual and thus enact appropriate behavior (Carré et al., 2009; Carré et al., 2010), for example that of avoidance.

The face seems also the basis on which we formulate judgments about people concerning aspects that should not be judged from the mere external appearance of someone.

The face seems to have an informative value even when choices should be based on other kinds of information (Zebrowitz and Montepare, 2008; Todorov et al., 2013). An individual whose face is judged as more criminal than another will more likely be judged as guilty (Flowe and Humphries, 2011) or a political candidate whose face inspires trustworthiness to be elected (Olivola and Todorov, 2010), or a person judged to be cold and incompetent could suffer from episodes of social exclusion (Rudert et al., 2017). Therefore, it seems reasonable to suppose that the perception of images, above all images of human faces, quickly analyzed on SNSs can facilitate the manifestation of phenomena such as the expression of a sentiment like hate, social discrimination, or cyberbullying regarding those perceived as weaker (Kuchta and Miklošík, 2017). Stereotypes can be activated from clues of even little significance, and once they have been summoned, they require nothing more than confirmation (Robinson et al., 1995): who is seen as a victim can be victimized, and who is perceived as an aggressor will more likely be treated as violent, as seen, for example, in gender roles in videogames (Vella et al., 2019).

However, which stereotypes are activated when viewing an image of a face might not depend only on the face characteristics. It is reasonable to assume that the perception of a face as belonging to a person with a story of aggression or victimization can also partially come from the characteristics of the person who perceives it (Digman, 1990; Mattarozzi et al., 2015). This hypothesis, however, has not yet been verified by empirical evaluations. To our knowledge no study has concomitantly considered the role that the personality characteristics of the observer and his or her empathic capabilities play on the manifestation of stereotypical victim/aggressor judgments based on the simple observation of faces, with neutral emotional expression, of young men and women.

## Theoretical Framework

### Elaboration of Social Judgments on Faces

Perceptual analysis of the face is one of the fundamental processes that makes up social cognition. Faces are the most extraordinary attractor of attention (Farroni et al., 2002) and their perception is fundamental for the regulation of social interactions, and in general for our survival in society.

The way in which we perceive an individual’s face allows us not only to define identity based on social categories (sex, age, ethnicity) but also to express judgments, or pre-judgments, regarding their personality (Zebrowitz and Montepare, 2008).

Just as the relative information on the stable characteristics of a face, the information and clues relative to facial expressions can be subject to an “overgeneralization effect” (Oosterhof and Todorov, 2009; Todorov et al., 2011). According to the ecological-evolutionist approach, the appropriate response to emotional expressions—like keeping one’s distance from an angry person and getting closer to a happy person—has, over the course of our evolution, generated a noteworthy ability to consequently respond even before faces that are emotionally neutral yet the facial structure itself recalls a particular emotion (Zebrowitz and Montepare, 2006).

Judgments and inferences that individuals make on the basis of facial characteristics seem to be ascribable to two principal dimensions: valence and dominance, “where valence evaluation is an overgeneralization of perception of facial cues signaling whether to approach or avoid a person, and dominance evaluation is an overgeneralization of perception of facial cues signaling the physical strength/weakness of the person” (Oosterhof and Todorov, 2008, p. 11091). Emotionally neutral faces perceived as happy or angry generate either positive or negative trustworthiness, whereas emotionally neutral faces seen as more or less masculine generate a judgment of dominance (interpreted as the ability of a person to inflict physical damage) (Flowe, 2012). Men with a more masculine look are therefore evaluated as less warm, caring, and romantic and more competent, while men with more feminine characteristics, having the so-called baby-face, are associated with less aggressiveness and evaluated as more paternally investing. This is coherent with a process of categorization that starts with the perception of relevant physical characteristics (e.g., more baby-face traits) activating the content of gender stereotypes corresponding to the traits inferred by the person (Perrett et al., 1998; Kruger, 2006).

A recent study (Freeman and Johnson, 2016) shows how stereotypes interfere in the formation of judgment from the very first phase of the perception process. According to the Dynamic Interactive (DI) model (Freeman and Ambady, 2011), a neural computational model of social perception, the perception of other people is the result of the integration of two processes: a bottom-up process of the evaluation of physical facial characteristics, and a top-down process of highly cognitive elaboration (stereotypes, inclinations, objectives). According to this model the face is defined based on a series of social categories like gender, ethnicity, and age; tightly correlated amongst themselves which activate certain stereotypes. The perception of specific physical characteristics always gives rise to the parallel activation of more social categories where stereotypes are initiated in variable measure (Stolier and Freeman, 2016).

### Subjective Factors in Face Perception

The judgments that individuals express based on the face of someone they do not know are also influenced by the gender of the observer and by his or her personality characteristics (Todorov et al., 2015).

Studies have shown that women tend to be better than men in recognizing unknown faces, particularly female faces, among other face-like stimuli, while the opposite seems to hold for men, although the existence of the so-called own gender bias in face recognition is still debated (Ryan and Gauthier, 2016). Further, women tend to judge unknown faces as more trustworthy (and therefore with characteristics of the baby-face type) than men (Mattarozzi et al., 2015).

Many of the studies on the influence of personality traits on social judgments refer to the Big Five model, which includes five different personality traits: extraversion, neuroticism, consciousness, agreeableness, and openness to experiences (Digman, 1990; Mattarozzi et al., 2015). In particular, the dimension of agreeableness seems to be positively correlated to perceived trustworthiness. This finding is consistent with what has been defined by the Big Five model, where low levels of agreeableness are associated with skepticism.

Another effect that has been documented is the trait-congruence effect (Perlman et al., 2009), in which individuals tend to orient their attention more on characteristics of the stimulus that are consistent with the personality traits that they themselves are characterized by. For example, individuals with a high score of neuroticism and who are particularly anxious are faster to respond than those who are less anxious, direct their attention toward negative social stimuli (a threatening face, for example), and tend to maintain their attention for longer on elements that are more emotionally significant (eyes) (Perlman et al., 2009).

Beyond personality traits, some studies have concentrated on the evaluation of empathic ability in the individuation of facial expressions, and on the role the different components of empathy can have (Davis, 1980; De Waal, 2008). Empathic abilities evidently have a role in recognizing emotions starting from the evaluation of facial expressions. Some studies, for example, have shown that individuals characterized by high levels of empathy have greater accuracy in recognition (Chikovani et al., 2015) and are faster (Kosonogov et al., 2015) with facial expressions that accompany emotional states.

### Problematic Internet Use and Aggressive Behavior Online

There is an increasing awareness of the positive socio-psychological effects that social media, increasingly popular and targeted for different users and types of use, can have both on individuals, particularly young ones, and society (Mäntymäki and Islam, 2016). The use of SNSs, however, does not only depend on the characteristics and availability of social media itself. It was highlighted that the choice of a specific social media can depend on the personality characteristics of the user (Marengo et al., 2020). Generally, the use of social media appears to be related to extraversion and, to a lesser extent, to conscientiousness and neuroticism.

From the individual point of view, sharing the emotional state of others, or on the contrary provoking emotional states in others through aggressive behavior online, can also depend on the modes of interaction that can be indicated as problematic (Casas et al., 2013). Intensive use of SNSs can be associated with multiple negative consequences. Perhaps the first among them is the intensification of depressive symptoms that can become progressively more serious (Sampasa-Kanyinga and Hamilton, 2015; Appel et al., 2016; Escobar-Viera et al., 2018; Ozimek and Bierhoff, 2019; Wartberg et al., 2020; Hussain et al., 2020).

Further, some years ago some studies showed that youngsters, who feel the greatest need to stay online, experience feelings of shortcomings, isolation, and anger as a consequence of being deprived of the possibility to interact online, and more easily begin to express behaviors that can be qualified as cyberbullying (Tsai and Lin, 2001; Ybarra and Mitchell, 2004).

More recently, a study involving more than 4,000 Korean students found that those who had been a victim of cyberbullying or were perpetrators of aggressive behavior online, or who have experienced both, also showed an increase in the problems of Internet use (Jung et al., 2014). It was also shown that there is a difference in the subjects who report to have behaved aggressively on SNSs and those who instead deny it. More particularly, some studies highlight how social media use exceeding 3 h a day can be sufficient in having a greater probability of falling into a state of nervousness and anxiety from Internet use deprivation (Park et al., 2013). It can further contribute to putting into place negative behaviors such as cyberbullying (Bracci et al., 2019; Parlangeli et al., 2019).

## The Current Study

The study reported in this paper was conducted to investigate a series of research questions, and test hypotheses at different levels of specificity. The first question addressed was whether the perception of high-resolution photographs of human faces, not characterized by any emotional expression, is able to cause the activation of stereotypes concerning past events of aggression, perpetrated or endured by the person whose face is viewed (RQ1). Asking participants to report their judgments about violent behavior by referring to the past vs. the present or future may have advantages. A large body of studies has in fact shown that imagining past events (in this case having had stories of aggression or victimization) and imagining future events (being able to become a participant in stories of aggression or victimization) leads to different results. In the first case, when referring to the past, emphasis tends to be placed on negative aspects, while the opposite is true when referring to things that have yet to happen (Ross and Newby-Clark, 1998; Newby-Clark and Ross, 2003; Berntsen and Jacobsen, 2008; Berntsen and Bohn, 2010). Therefore, in order to bring out more explicitly hypothetical negative stereotypes such as aggressor and victim, in this study it was preferred to ask questions that referred to a possible past of aggression or victimization, rather than focusing on whether the faces assessed were potentially referable to people who might now or in the future become aggressors or victims.

To qualify the first research question, in light of what has been pointed out in literature (Kruger, 2006; Marwick and Boyd, 2014), we hypothesized that male faces more easily activate stereotypical associations tied to aggressive behavior than female faces (H1), and secondly that judgments can be different when expressed by men on male faces and women on female faces and vice versa (H2). We also wanted to verify the existence of the “aggressor stereotype,” as masculine and strong, and the “victim stereotype” as feminine and weak (H3) (Stolier and Freeman, 2016).

The second research question was whether some subjective characteristics, such as personality traits and empathic abilities, can moderate (facilitate or impede) the perception of individuals as more or less aggressive, more or less strong, and more or less subject to being a victim (RQ2) (Mattarozzi et al., 2015) when observing faces with emotionally neutral expressions. Previous research has shown that individuals with a high level of neuroticism tend to allocate more attention to facial stimuli that show an expression of threat (Perlman et al., 2009). It can thus be hypothesized that the trait of neuroticism can lead to perceiving even neutral faces as more aggressive (H4), and this could also be connected with the gender of the observer and/or that of the face observed (H5) (Becker et al., 2007; Willis et al., 2013).

With regard to empathic abilities, higher empathy may lead to greater sensitivity in recognizing facial expressions of negative emotions such as fear (Chikovani et al., 2015). Thus, it can be hypothesized that the tendency to attribute victimization experiences is stronger by observers who have higher empathy (H6).

Finally, it is worth exploring the relations between SNSs use and the activation of victims/aggressor stereotypes. In particular, time spent interacting with social media appears to be related to neuroticism (Chow and Wan, 2017). Therefore, we can hypothesize that massive use of social media is related to the activation of stereotypes pertaining to the experience of offensive behavior perpetrated or suffered (H7).

## Materials and Methods

### Participant and Procedure

One hundred and eighty-five subjects participated in the study aged between 14 and 26 years, 56.2% female (104) and 43.8% (81) male, with a median age of 18.84 (*SD* = 3.6, minimum 14 maximum 26, *M* = 18.78, *SD* = 3.7; *F* = 18.88, *SD* = 3.5). Students represented 94.6% (55.1% high school students and 39.5% university students) and workers represented 5.4%. Nearly all participants interacted with at least one form of social media daily. In particular, 19.5% use it for more than 3 h a day (*M* = 16.0%; *F* = 22.1%), 50.8% from 1 to 3 h a day (*M* = 45.7%; *F* = 54.8%), 27.6% use it less than 1 h a day (*M* = 35.8% *F* = 21.2%), and 2.2% claim to never use it (*M* = 1.2% *F* = 1.0%).

Participants were asked to answer a specifically structured online questionnaire.

The university students and workers who participated in the study completed the questionnaire inside the Laboratory of Psychological Experimentation of the Dispoc (Dipartimento di Scienze Sociali Politiche e Cognitive) of the University of Siena after having given oral consent. The subjects who were recruited to take part in the experiment within the departmental premises were contacted by the experimenter (MB or EM) without any selective procedure. The experimenter waited at the entrance of the department and asked those who entered if they were willing to take part in an experiment on the perception of human faces. The potential participants were contextually informed that the experiment would last about 20–30 min and that the questionnaire contained questions about their personality. Each person who agreed to take part in the experiment was invited to the psychology laboratory where he or she was informed of the objectives of the study, all the questions it contained, and the possibility of interrupting the compilation at any time. None of the subjects stopped filling in the questionnaire and none of them reported feeling fatigued when completing it.

High school students completed the questionnaire in their own class in the presence of one of the research team members. Students were invited to take part in the study, on a voluntary basis and after headteachers and family authorizations. They too, before the compilation began, were informed about the objective of the study, the questions in the questionnaire, the expected duration of the compilation, and that they could interrupt the compilation at any time. All the high school participants completed the questionnaire and none of them made any remarks about the length of time needed to finish.

All the participants in the study were informed that their data would be treated in an aggregated manner and that they had no fixed time to fill in the questionnaire. No recompense was offered for participation in the study.

The department, which carries out the function of ethical committee in our case, evaluated and approved the study (September 27, 2017; report no.10/2017).

### Experimental Material

#### The Questionnaire and the Scales

The data were gathered from a specifically structured questionnaire to be filled in online^1^. The questionnaire was made up of 157 [7 + 20 + 10 + 120 (30^∗^4)] questions and divided into 4 sections.

The first of these was directed at gathering general information on the sample group and on personal data (age, gender, student/worker) and on the type and frequency of social networks used (7 questions).

The second (20 questions) aimed at examining the subject’s level of empathy through the BES Basic Empathy Scale (Italian version in Albiero et al., 2009). The third (10 questions) aimed at revealing personality traits with the Big Five Personality Test (Gosling et al., 2003). The last section (with a total of 120 questions) contained 30 faces, 15 men and 15 women, selected from a free database of face stimuli, the Chicago Face Database (CFD version 2.0.2-March 2016; Ma et al., 2015).

Each subject saw the face stimuli selected in sequence and could see each face for as long as they liked. For each face four questions were posed, and evaluation of the face was expressed on a five-point scale regarding: the level of masculinity/femininity (How does this face look? 1 “masculine”–5 “feminine”), weakness/strength (Can you sense if this person is 1 “very weak”–5 “very strong”), the possibility that the person could have been the victim or perpetrator of mistreatment over the course of their lives (Do you think that this person could have endured past mistreatment? 1 “absolutely not”–5 “absolutely yes,” Do you think that this person, in the past, could have had aggressive behavior toward other people? 1 “absolutely not”–5 “absolutely yes”). The face that was evaluated was always displayed while the participant answered the four questions concerning it.

#### The Basic Empathy Scale

In order to measure the level of empathic responsiveness, an Italian version of the BES Basic Empathy Scale (Jolliffe and Farrington, 2006; Albiero et al., 2009) was used, which defines empathy as a multi-dimensional concept. This includes a cognitive dimension, CE (cognitive empathy), interpreted as the ability to recognize and understand the emotions of others, and an affective dimension, AE (affective empathy), interpreted as the suitable emotional response to the emotions of others.

The Italian version, like the English one (Jolliffe and Farrington, 2006), is made up of 20 questions and includes 11 items to reveal AE (item 1^∗^, 2, 4, 5, 7^∗^, 8^∗^, 11, 13^∗^, 15, 17, 18^∗^) and 9 items to reveal CE (item: 3, 6^∗^, 9, 10, 12, 14, 16, 19^∗^, 20^∗^). The scores of the items indicated with an ^∗^ have to be reversed (e.g., 1 becomes 5). For each item individuals were asked to express their level of agreement on a scale akin to the Likert scale from 1 “never true” to 5 “always true” (e.g., “A friend’s emotions do not touch me much” 1 “never true”–5 “always true”). The points relative to the two subscales and the overall points were calculated by adding up the two subscales (BTS BesTotalScore) for each individual.

#### The Big Five Questionnaire

For personality evaluation, a 10-item short version of the Big Five translated into Italian was used (Gosling et al., 2003; Rammstedt and John, 2007; Guido et al., 2015). The scale defines personality based on five main traits: extraversion (items, 1^∗^,6), agreeableness (items 2, 7^∗^), conscientiousness (items 3^∗^, 8), neuroticism (items 4, 9^∗^), and openness to experience (5^∗^, 10). For each trait the scale identifies two items that define opposite concepts (e.g., for the extraversion aspect: “I see myself as a reserved person,” “I see myself as a person who is social and at ease”). For each item the individuals are asked to express their level of agreement on a Likert-type scale from 1 “do not agree” to 5 “completely agree.” The items indicated with an “^∗^” are reverse questions.

#### The Stimuli: Chicago Face Database

Thirty high-resolution photograph images were presented to the participants that show both male (*n* = 15) and female faces (*n* = 15). Three different orders of image presentation were created to avoid a “presentation sequence” bias. The face stimuli were selected from the Chicago Face Database (CFD version 2.0.2—March 2016) (Ma et al., 2015), free facial stimuli of 597 high-resolution, standardized photographs of black and white men and women of varying ethnicity (Asian, Black, Latino, White) between the ages of 18 and 40. For each face there are extensive data including both physical attributes (e.g., face width, nose shape.) as well as subjective ratings by independent judges (e.g., attractiveness, masculinity, femininity…). The database includes photographs with varying facial expressions: neutral, angry, fear, happy with closed mouth, happy with open mouth.

**FIGURE 1:**
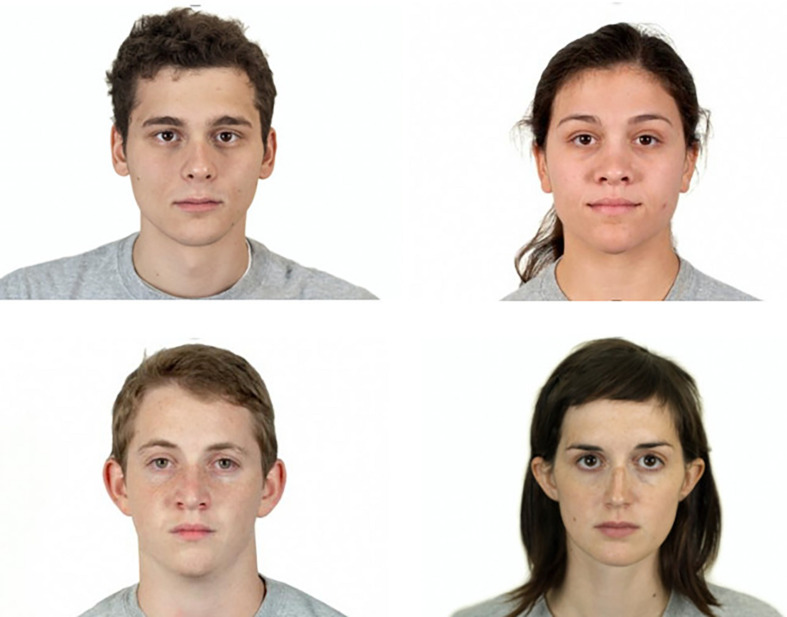
Four of the face stimuli extracted from the Chicago Face Database (Ma et al., 2015) considered in the study.

For our study we chose faces that were homogeneous in their ethnicity (White) and considered neutral (N) in emotional expressiveness. The average age attributed to the faces by the participants of the CFD study was 22.31, *SD* = 1.57, whereas the averages for attractiveness (3.37, *SD* = 0.67), dominance (2.40, *SD* = 0.55), trustworthiness (3.46, *SD* = 0.35), and perceived racial prototypicality (3.57, *SD* = 0.78) relate to a scale from 1 (not at all) to 7 (extremely), and are all relevant aspects particularly influential for our study (see Figure 1).

Male and female faces significantly differed only in age (average age, M + F = 22.31; males = 21.35, *SD* = 1.64; females = 23.26, *SD* = 0.77; *t* = 1,707, df = 28, *p* < 0.000).

### Statistical Analysis

To investigate the research questions and test the research hypotheses that we have previously presented, different statistical analyses were conducted. Descriptive statistics were initially computed about the characteristics of the sample (demographic information, personality traits and empathy, social network use) and compared by gender using *t*-tests or Chi-square tests.

The main statistical technique used to analyze the judgments about the faces were linear mixed-effects models (LMMs), which are an extension of the linear model behind linear regression and ANOVA, and are able to model multiple sources of random variability simultaneously (Baayen et al., 2008). Since the faces were actually a random sample of all the possible faces we could have used in the experiment, including this last source of random variability in the model is important to allow generalizability of the results. To test whether each random effect in the model (a variance parameter) significantly contributed to increasing the models’ fit, we compared models including different combinations of random effects using likelihood ratio tests (for models nested in each other), and information measures (AIC, BIC) (for non-nested models). *P*-values for main effects and interactions, and related approximate degrees of freedom, were computed with Satterthwaite’s method (Fai and Cornelius, 1996).

To test H1, H2, and H3, we used observer gender, face gender, and their interaction, as predictors of the ratings of the faces. Different models were fit, each using a different type of rating as dependent variable. To test H4, H5, and H6, further models were fitted including the measures of empathy and personality traits as predictors of the ratings of a past of aggression as victim or perpetrator. To test the effect of these variables on the ratings, they were entered in the models both individually and simultaneously. To test the role of these variables in the differences between men and women in the use of stereotypes, each measure of empathy or personality was included in two-way and three-way interactions with observer gender and face gender.

To investigate the relationship between social media use and empathy and personality traits we used a MANOVA including time on social networks as the independent variable, and all the measures of empathy and personality as dependent variables. To test the possible relationships between the ratings of faces and social media use (H7), we used LMMs including time on social networks as a predictor of the ratings of aggressor or victim, alone or in interaction with observer gender and face gender.

To test the relationships between the ratings of faces along the different dimensions, and thus investigate further the nature of the aggressor and victim stereotypes (H3), we used multilevel (partial) correlations, which are partial correlations based on LMMs that include grouping factors as random effects to control for random individual variability and account for the repeated measures collected for each participant (Makowski et al., 2020). Given the high number of observations used in the analysis (*N* = 5,550), we decided to report only the correlations of at least moderate strength (| r| > 0.3).

All the statistical analyses were computed using the R statistical computing software (v. 4.0.2), and the functions in *lme4* (v. 1.1-25, Bates et al., 2015) and *correlation* (v. 0.6.0, Makowski et al., 2020).

## Results

The data analysis tried to highlight evaluations of the sample considered as a whole, but also to point out the differences between female and male participants regarding different levels of empathic ability, personality characteristics, and interactions with social media.

### Gender Differences and Stereotypes

To verify the frequencies of judgments that attributed a history of aggression to the persons represented in the faces, either as victim or as perpetrator, we initially computed for each participant the percentage of the faces of the men and women that were judged as those of aggressors or of victims. The percentages were then averaged by observer gender and face gender, collapsing across participants. The results are presented in Table 1. A past of aggressor was attributed to *male faces* on average between 29.5% (judgments by women) and 36.7% (judgments by men) of the times, and to *female faces* between 24.2% (judgments by women) and 26.5% (judgments by men). Conversely, a past of being a victim was attributed to *female faces* between 39.7% (judgments by women) and 43.1% (judgments by men), and to *male faces* between 30.4% (judgments by men) and 32.1% (judgments by women).

**TABLE 1:** Average percentage of male and female faces perceived as having been aggressors and victims by male and female observers.

Observer gender	Face gender	Aggressor		Victims	
		*Mean*	*Sd*	*Mean%*	*Sd%*
Female	Female	24.2%	17.4%	39.7%	21.4%
Female	Male	29.5%	19.2%	32.1%	18.8%
Male	Female	26.5%	19.4%	43.1%	20.0%
Male	Male	36.7%	21.9%	30.4%	19.9%

**FIGURE 2:**
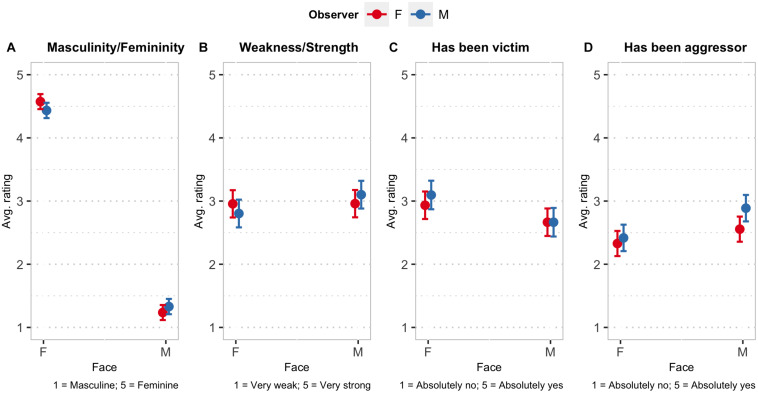
Marginal means of the ratings of the faces concerning **(A)** masculinity/femininity, **(B)** weakness/strength, **(C)** having been a victim of acts of aggression in the past, and **(D)** having been perpetrators of acts of aggression in the past, as a function of the gender of participants and of the gender of the people represented in the faces. Error bars are 95% confidence intervals of the means, estimated from the fitted LMMs.

Linear mixed-effects models (LMMs) were used to analyze the ratings of the faces, using observer gender (i.e., gender of the participant), the face gender (i.e., the gender of the person in the face), and their interaction as predictors, and the ratings of masculinity/femininity, of weakness/strength, and of the believes that the person represented had been a victim or perpetrator of aggressive acts as dependent variables (in separate analyses). The marginal means of the ratings for all the variables are plotted in Figure 2 as a function of observer and face.

The results of the analysis of the masculinity/femininity ratings revealed a significant main effect of face [*F*_(__1, 28.1)_ = 1529.3, *p* < 0.001] and a significant observer by face interaction [*F*_(__1__, 5272__.0)_ = 36.6, *p* < 0.001]. The marginal means of the ratings as a function of observer and face are presented in Figure 2A. Not surprisingly, female faces were rated as significantly more feminine than male ones [mean difference = 3.22, *z* = 39.1, *p* < 0.001]. The analysis of the simple contrasts, moreover, showed that female faces were rated as significantly more feminine by female observers than by male ones [mean difference = 0.14, *z* = 4.21, *p* < 0.001] while male faces were rated as significantly less feminine by females than by males [mean difference = −0.09, *z* = −2.83, *p* < 0.01].

The LMM analysis of the weakness-strength ratings uncovered a significant observer by face interaction [*F*_(__1__, 5230__.3)_ = 32.5, *p* < 0.001] and no significant main effects. The analysis of the simple contrasts revealed a clear own-gender effect: female faces were rated as significantly stronger by women than by men [mean difference = 0.15, *z* = 2.9, *p* < 0.01] while male faces were rated as significantly stronger by men than by women [mean difference = 0.14, *z* = 2.69, *p* < 0.01], as it can be seen in the plot of the marginal means in Figure 2B.

The analysis of the ratings about the belief that the person behind the face had been a victim of aggression also showed a significant main effect of face [*F*_(__1, 28.0)_ = 6.54, *p* < 0.05] and a significant interaction [*F*_(__1__, 5234__.1)_ = 8.75, *p* < 0.01]. The ratings were first of all significantly higher for female faces than for male ones [mean difference = 0.35, *z* = 2.6, *p* < 0.05]. As it can then be seen in Figure 2C, for faces of men, the ratings given by female and male observers were on average almost exactly the same, while for female faces the ratings given by women were slightly lower than those expressed by male observers, although the difference was not statistically significant [mean difference = −0.16, *z* = −1.85, *p* = 0.065].

Finally, the analysis of the ratings about having been an aggressor highlighted significant main effects of both observer [*F*_(__1, 183.5)_ = 5.74, *p* < 0.05] and face gender [*F*_(__1, 28.1)_ = 8.7, *p* < 0.001], and a significant interaction among the factors [*F*_(__1__, 5233__.7)_ = 19.34, *p* < 0.001]. The marginal means as function of observer and face are plotted in Figure 2D. Male faces were rated more like those of aggressors than female faces. Male faces, moreover, were rated more like those of aggressors by male observers than by female ones [mean difference = 0.33, *z* = 3.61, *p* < 0.001]. For female faces, instead, ratings by male and female observers were not significantly different [mean difference = 0.09, *z* = 0.96, *p* = 0.34]. Moreover, male observers rated male faces as significantly more aggressive than female faces [mean difference = 0.47, *z* = 3.85, *p* < 0.001], while female observers did not rate male faces as significantly more aggressive than female faces, although numerically the average ratings for male faces were also higher than the ones for female faces [mean difference = 0.23, *z* = 1.88, *p* = 0.06].

**FIGURE 3:**
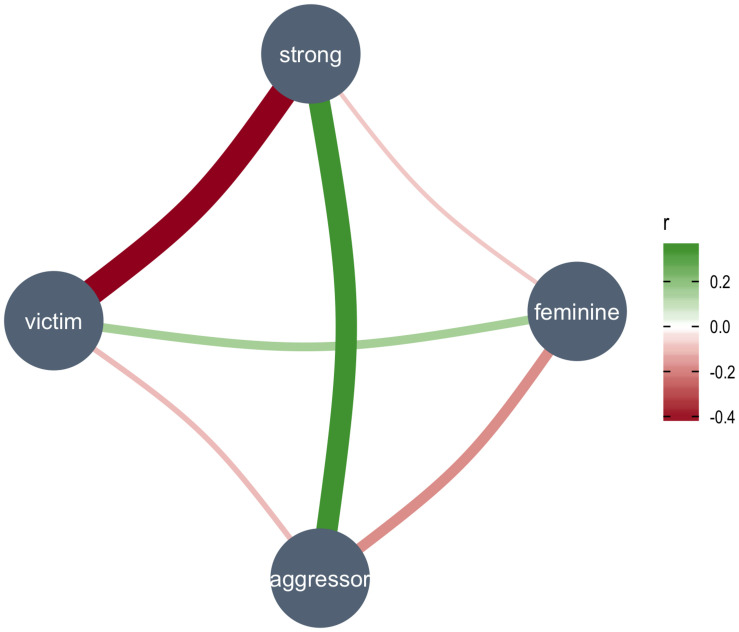
Plot of the multilevel (partial) correlations among the judgments about the faces in the form of a Gaussian graphical model. The thickness and color of the links represent the size and direction of the partial correlations.

To further understand the relationships between the different types of judgments, and to try to characterize the nature of the stereotypes of aggressors and victims, we computed multilevel (partial) correlations. To estimate the correlations among the ratings, adjusted for individual variability, we included participant id as a random factor in the analysis. The results of the analysis showed that all the correlations were statistically significant, but only two correlations met the size criteria that we had chosen for reporting (| r| > 0.3), and both involved the ratings of the face along the weakness-strength dimension which were positively correlated with the ratings of having been an aggressor [*r* = 0.35, *t*(5,409) = 27.9, *p* < 0.001], and negatively with the ratings of having been the victim of aggression [*r* = −0.45, *t*(5,409) = −35.5, *p* < 0.001]. To visualize the relationships among the judgments we used Gaussian graphical models (GGM) (Bhushan et al., 2019), in which the partial correlations among the variables are plotted as a graph (Figure 3). A GGM plot includes a set of variables represented as circles (“nodes”), and a set of lines that visualize relationships between them, whose thickness represents the strength of association between the variables.

### Personality Traits and Empathy

In the table below (Table 2) the average values of the scores of men and women on the variables measuring empathic abilities and personality traits are reported. On the empathy measures, male participants had significantly lower scores than female ones, a result which is consistent with previous studies (Davis, 1983; Baron-Cohen and Wheelwright, 2004; Rueckert and Naybar, 2008). Concerning personality traits, male observers had significantly lower scores on neuroticism than women, another result consistent with reports in the literature (Chapman et al., 2007); the male faces result as more emotionally stable and less fragile from an affective point of view.

**TABLE 2:** Average scores on the measures of empathy and personality traits, overall and by gender.

	All	*F*	*M*	*P*-value
	*N* =184	*N* = 104	*N* = 80	
Affective empathy	3.27 (0.53)	3.45 (0.52)	3.03 (0.43)	**<0.001**
Cognitive empathy	3.94 (0.50)	4.02 (0.48)	3.84 (0.50)	**0.018**
Empathy (BES total score)	3.71 (0.46)	3.86 (0.41)	3.51 (0.45)	**<0.001**
Extraversion	3.18 (1.02)	3.10 (1.14)	3.29 (0.84)	0.202
Agreeableness	3.05 (0.79)	3.00 (0.82)	3.11 (0.75)	0.337
Consciousness	3.40 (0.90)	3.47 (0.87)	3.32 (0.93)	0.288
Neuroticism	3.28 (1.11)	3.61 (1.01)	2.86 (1.09)	**<0.001**
Openness	3.63 (0.95)	3.73 (0.93)	3.49 (0.98)	0.097

*P-values (bold values are significant) derive from independent sample t-tests comparing the average scores for each variable across genders.*

### Stereotypes, Empathy, and Personality Traits

To investigate the relationships between personality traits and judgments about the faces, we initially computed, for each participant, the average ratings for each dimension (weakness/strength, has been victim, has been aggressor) collapsing across faces. These scores can be seen as measures of individual participants’ tendencies to express judgments of strength, and about how being a victim or aggressor can be represented in a face. The masculinity/femininity dimension was excluded because averaging across faces of different gender, the ratings for male and for female faces would cancel each other, resulting in a misleading estimate. We then computed the correlations between the average ratings and the empathy and personality variables.

The results showed that the attribution of aggressive behaviors was negatively correlated with affective empathy (*r* = −0.212, *p* < 0.01) and BES total empathy (*r* = −0.171, *p* < 0.05). However, when the correlation analysis was repeated separately for male and female participants, the results showed than only for women there was a significant, inverse correlation between affective empathy and judgments about aggressivity (*r* = −0.226, *p* < 0.026).

To test the possible association of empathic abilities and personality traits with the stereotypes activated by faces, we first used univariate and multivariate linear mixed-effects regression models. In the univariate models, the different variables measuring empathy and personality traits were used individually as predictors of the ratings (about whether the person represented in the face had been in the past perpetrators or victims of aggressive acts). In the multivariate models, all the personality and empathy variables were used simultaneously as predictors. In all the fitted models, we included two random effects, relative to the faces and the observers.

The results showed that the ratings of whether the person represented in the faces had been a victim of aggression were not significantly associated with any of the variables concerning empathic abilities or personality traits, neither individually nor in the additive multivariate model. The ratings about having been an aggressor, instead, were significantly and negatively associated with BES affective empathy and BES total, both in the univariate models [affective empathy: *B* = −0.23, *F*_(__1, 175.6)_ = 7.5, *p* < 0.01; BES total empathy: *B* = −0.21, *F*_(__1, 173.5)_ = 4.61, *p* < 0.05] and in the multivariate ones [affective empathy: *B* = −0.25, *F*_(__1, 165.3)_ = 6.39, *p* < 0.05; BES total empathy: *B* = −0.24, *F*_(__1, 165.0)_ = 5.12, *p* < 0.05]. No other variable or personality trait was significantly associated with the aggressor ratings, neither in a univariate or in a multivariate model.

We then fitted further multivariate mixed-effects regression models, including all the individual empathic and personality variables (in separate models, one with empathic variables and one with personality traits) and their interactions with observer gender and face gender as predictors of the judgments about the faces. In this way we could test the moderator effect of empathy and personality traits on stereotypical judgments, and investigate whether and how these variables could contribute to explain the results from the linear mixed-effects models analysis of the gender differences and stereotypes.

**FIGURE 4:**
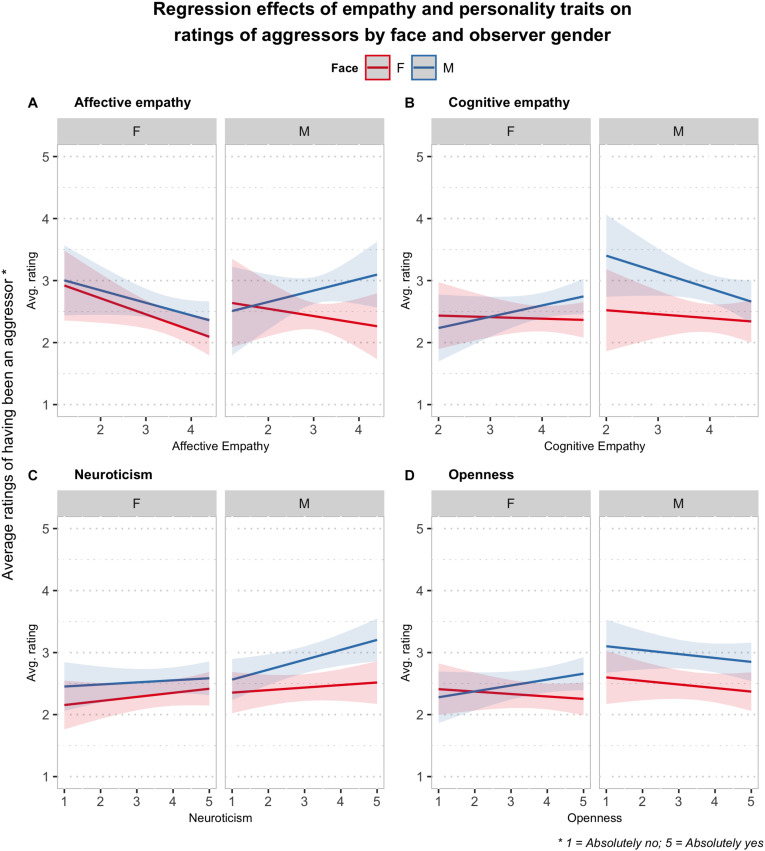
Plots of the three-way interaction effects (i.e., regression slopes) estimated from the linear mixed-effects models including empathy **(A)** affective, **(B)** cognitive and personality variables **(C)** neuroticism, **(D)** openness to experience as predictors of the ratings about the history of aggression for the persons represented in the faces.

The results of the analysis of the effect of empathy on the ratings of aggressors showed significant two-way interactions between face gender and observer gender [*F*_(__1__, 4970__.4)_ = 4.34, *p* < 0.05] and between face gender and affective empathy [*F*_(__1__, 4970__.5)_ = 7.22, *p* < 0.01], a significant three-way interaction between cognitive empathy, face gender, and observer gender [*F*_(__1__, 4970__.4)_ = 10.97, *p* < 0.001], and a marginally significant three-way interaction between affective empathy, face gender, and observer gender [*F*_(__1__, 4970__.5)_ = 3.35, *p* = 0.067]. In Figure 4, the predicted ratings as a function of observer gender, face gender, and (along the *x*-axis) affective (A) or cognitive (B) empathy are plotted, in which the nature of the significant, three-way, interaction effects are evident. For female observers, in fact, affective empathy had a significant negative effect on the ratings of aggressors concerning female faces (*B* = −0.26, 95% CI: [−0.49, −0.02]), but not male faces (*B* = −0.20, 95% CI: [−0.44, 0.03]). In male observers, instead, a similar effect was not found on the judgments neither about female faces nor male ones, but a test of the interaction contrast showed that the effect of empathy on the ratings (i.e., the slope) was significantly higher for male faces than for female faces (Ψ*_*F*__–__*M*_* = −0.30, *z* = −2.68, *p* < 0.01). Cognitive empathy, moreover, in male observers seemed to decrease the ratings about an aggressive past for male faces (*B* = −0.27, 95% CI: [−0.58, 0.05]) and had basically no effect on the corresponding ratings for female faces (*B* = −0.06, 95% CI: [−0.38, 0.25]). In female observers moreover cognitive empathy did not seem to influence the ratings of female faces (*B* = −0.02, 95% CI: [−0.27, 0.23]), but tended to increase the ratings of aggression for male faces (*B* = 0.18, 95% CI: [−0.07, 0.43]). For both male and female observers, moreover, the regression slopes (i.e., the effect of empathy on the ratings) for empathy varied significantly across face genders (female observers: Ψ*_*F*__–__*M*_* = −0.21, *z* = −2.72, *p* < 0.01; male observers: Ψ*_*F*__–__*M*_* = 0.20, z = −2.07, *p* < 0.05).

Concerning the effect of the personality traits on the aggressor ratings, the results of the analysis showed a significant two-way interaction between face gender and openness [*F*_(__1__, 5093__.5)_ = 4.54, *p* < 0.05], a significant three-way interaction between neuroticism, face gender, and observer gender [*F*_(__1__, 5094__.0)_ = 7.26, *p* < 0.01], and a significant three-way interaction between openness, face gender, and observer gender [*F*_(__1__, 5093__.6)_ = 5.40, *p* < 0.05]. The plots in Figure 4 of the interaction effects involving neuroticism (C) show first of all for men a negative trend in the effect of this personality trait (*B* = 0.16, 95% CI: [0.03, 0.29]) similar to the one of affective empathy on ratings of male faces, and no effect on female faces ratings, or for male observers. Concerning openness to experience the plots show (Figure 4D) that this trait has basically no effect on ratings of aggressors for males. For female observers, neuroticism also seemed to have no effect for judgments of female faces, but ratings for male faces tended to slightly increase with openness (*B* = 0.10, 95% CI: [−0.04, 0.23]), and the regression slopes differed significantly across face genders (Ψ*_*F*__–__*M*_* = −0.13, *z* = −3.34, *p* < 0.001).

**FIGURE 5:**
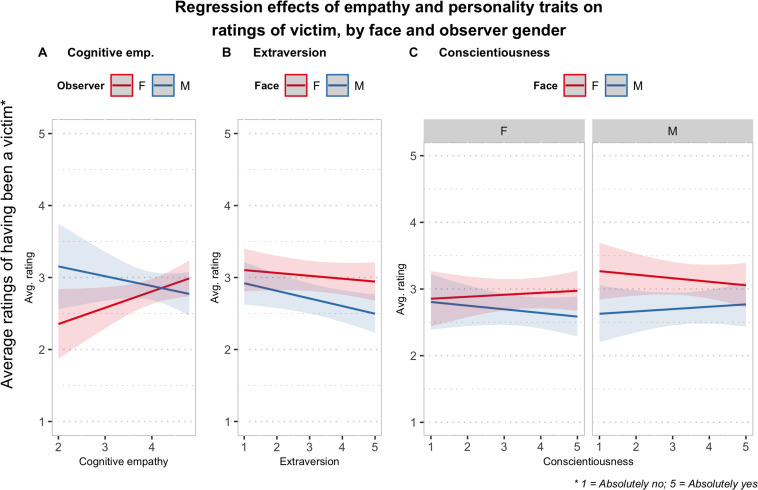
Plots of the two-way and three-way interaction effects (i.e., regression slopes) estimated from the linear mixed-effects models including empathy **(A)** affective and personality variables **(B)** Extraversion, **(C)** conscientiousness as predictors of the ratings about the history of having been a victim of aggression for the persons represented in the faces.

The same analyses were conducted on the ratings about having been a victim. It is interesting to notice, first of all, that when interactions with empathy measures or personality traits were included, the two-way interaction between face gender and observer gender was no longer significant. The results of the model that included affective and cognitive empathy, as covariates in interaction with gender and face, showed only a marginally significant interaction between cognitive empathy and observer gender [*F*_(__1, 169.7)_ = 3.83, *p* = 0.052]. The analysis of the simple trends (Figure 5A) revealed that cognitive empathy significantly increased the ratings of having a history as a victim for female observers (*B* = 0.23, 95% CI: [0.00, 0.45]). The results of the analysis with the personality traits covariates revealed a significant three-way interaction between conscientiousness, face gender, and observer gender [*F*_(__1__, 5094__.4)_ = 7.39, *p* < 0.01], and a significant two-way interaction between extraversion and face gender [*F*_(__1__, 5094__.2)_ = 5.45, *p* < 0.05]. The analysis of the interaction contrasts showed that none of the simple regression slopes were significant, but they showed opposite trends in men and women in the effect of conscientiousness (Figure 5C). Concerning extraversion, the results showed for men a significant negative effect on the ratings (*B* = −0.12, 95% CI: [−0.22, −0.02]), and no significant trend for female observers (Figure 5B).

### Stereotypes and Time on Social Media

It seems necessary to first clarify that when the analysis was conducted, in 2018, a rather differentiated, although pervasive, level of social media use was reported. Almost the entirety of the sample interacted on one or more social networks daily, also for multiple hours a day (all except 2). Table 3 reports the percentage of use of the different social networks in the sample.

**TABLE 3:** Use of different social media platforms in the overall sample and by gender.

	All	*F*	*M*	*P*-value
	*N* = *185*	*N* = *104*	*N* =81	
WhatsApp	176 (95.1%)	99 (95.2%)	77 (95.1%)	1.000
Facebook	140 (75.7%)	75 (72.1%)	65 (80.2%)	0.269
Instagram	132 (71.4%)	82 (78.8%)	50 (61.7%)	**0.017**
YouTube	131 (70.8%)	67 (64.4%)	64 (79.0%)	**0.045**
Google	101 (54.6%)	55 (52.9%)	46 (56.8%)	0.704
Snapchat	42 (22.7%)	26 (25.0%)	16 (19.8%)	0.504
Twitter	11 (5.95%)	6 (5.77%)	5 (6.17%)	1.000
Ask.fm	10 (5.41%)	5 (4.81%)	5 (6.17%)	0.750
Tumblr	9 (4.86%)	6 (5.77%)	3 (3.70%)	0.733
Pinterest	8 (4.32%)	7 (6.73%)	1 (1.23%)	0.081
Yahoo answers	7 (3.78%)	5 (4.81%)	2 (2.47%)	0.470
LinkedIn	3 (1.62%)	1 (0.96%)	2 (2.47%)	0.582
Telegram	1 (0.54%)	0 (0.00%)	1 (1.23%)	0.438

*The P-values (bold values are significant) reported are derived from Chi-squared tests conducted to compare frequencies in male and female respondents.*

Gender differences in social media interaction highlight that women, on average, mainly interacted with two forms of social media compared to men, specifically YouTube and Instagram. In fact, 50 male subjects out of 81 (61.7%) reported their use of Instagram, while 82 out of 104 women reported their use (78.8%) (χ^2^ = 6.527, *p* < 0.011). As far as YouTube is concerned, 64 men in a subsample of 81 (79%) used it whereas 90 out of 104 women said they used it (86.5) (χ^2^ = 4.689, *p* < 0.030). As for the time spent on various social media platforms (less than 1 h a day; from 1 to 3 h a day; more than 3 h), no differences between men and women were found.

**FIGURE 6:**
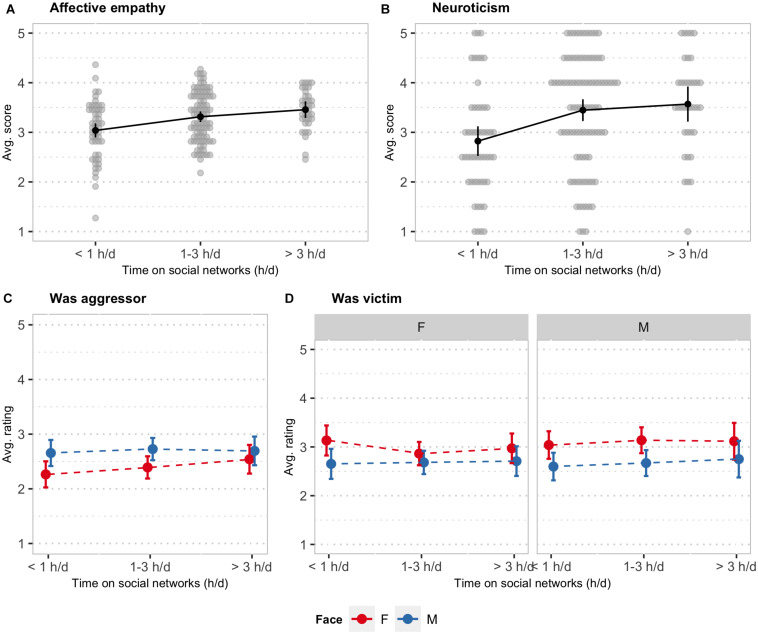
Plot of the marginal means of **(A)** affective empathy, **(B)** neuroticism, **(C)** ratings of a past of aggressor, and **(D)** ratings of a past as a victim, as a function of time spent on social media **(A–D)** and **(C)** face gender or **(D)** face and observer gender. In **(A)**, a continuous variable, and **(B)**, a discrete variable, the gray circles are individual data points. Error bars are 95% confidence intervals for the means.

To test the relationships between the time spent interacting on social media, personality, and empathy, we conducted a MANOVA including as the factor the time spent on social networks, and all the personality and empathy measures as dependent variables. The result of the multivariate test showed a significant effect of time on social networks [*F*_(__14, 326)_ = 2.65, *p* < 0.01] on the combined dependent variable. The results of the univariate tests showed significant differences by time on social networks on affective empathy [*F*_(__2, 168)_ = 10.05, *p* < 0.001] and neuroticism [*F*_(__2, 168)_ = 7.17, *p* < 0.01]. As it can be seen in the plots of the marginal means in Figures 6A,B, participants that reported to spend more time on social media had higher empathy and neuroticism.

We then used linear mixed-effects models to test whether the time spent on social networks was associated with judgments about aggressors and victims. The results showed that time on social networks did not have a significant effect on the ratings for victims or aggressors. Finally, we refit the models including the three-way interaction between time on social networks, observer gender, and face gender. The results showed a significant two-way interaction between time on social networks and face gender [*F*_(__2__, 5114__.6)_ = 3.97, *p* < 0.05] on aggressor ratings (Figure 6C), and a significant three-way interaction between time on social networks, face, and observer gender [*F*_(__2__, 5114__.2)_ = 3.25, *p* < 0.05] on victim ratings (Figure 6D). The analysis of the simple trends in the ratings of a past of aggression by time on social networks showed a significant positive linear trend in the ratings of female faces (*B* = 0.28, *z* = 1.99, *p* < 0.05), but not in the ratings of male faces.

## Discussion

Overall, the results of the analyses of the ratings highlighted significant differences in the judgments and perceptions of male and female faces. Female faces were judged not only significantly—and obviously—as more feminine than male ones, but also more as faces of people who had been victims of acts of aggression and less as faces of people who had been perpetrators of aggressive acts (Perrett et al., 1998; Kruger, 2006). We can assume that these results highlight a reference to gender stereotypes (Figure 2). Besides this, for each dependent variable, the analysis revealed a significant observer gender by face gender interaction. Both genders, in other words, attributed to faces of their own gender their positive stereotypical attributes more than they did for their opposite gender. Concerning the judgments of aggressors and victims, the analyses of the simple effects revealed that male observers tended to judge male faces more as those of aggressors than female observers, and that, vice versa, female observers tended to rate female faces less as those of victims than male observers (H1, H2, and H3). These results as a whole, suggest that the characteristic of having a past of aggression is part of the male stereotype (Harris and Knight-Bohnhoff, 1996; Mesquida and Weiner, 1996; Osman, 2011) and that in view of the shift toward a positive value of characteristics pertaining to one’s gender, it is also viewed by men as less negative than it is by women.

The fourth hypothesis (H4) is only partially supported by our results. The expression of judgments of past aggressive behavior tended to slightly increase with participants’ neuroticisms, although the trend was not significant. However, a much steeper, and statistically significant, trend was found in judgments by male observers concerning male faces, and the significant interaction supports the hypothesis (H5) that the effect of neuroticism is gender- and stimulus-specific.

The results revealed other interesting interactions between personality traits and judgments of aggression/victimization that had not been predicted. For female observers, ratings for male faces tended to slightly increase with openness, in a trend that is contrary to what happens with female faces. And when judgments are regarding a past as a victim, the trait of extraversion appears to have a different influence for male observers than for female observers. In fact, men, in comparison to women, make judgments that, as extraversion increases, consider fewer possible victimization experiences. In addition, when the possible effect of conscientiousness is considered, it is possible to highlight an opposite trend in men and women for what concerns victimization judgments.

Regarding the hypothesis about the relationship between empathy and judgments considering a past of victimization (H6), this seems to hold only for female observers. As cognitive empathic skills increase, female observers show a significant increase in judgments about past experiences of victimization, while for men a non-significant negative trend is observed. Whereas a much more complex picture emerges with respect to judgments of aggressions perpetrated in the past. For female observers, in fact, affective empathy had a significant negative effect on the ratings of aggressive behavior, but for men this negative trend was only observed for judgments of female faces, while ratings of male faces tended to increase with affective empathy. Cognitive empathy, moreover, in male observers seemed to decrease the ratings about an aggressive past for male faces, but to increase the same judgment by female observers for males faces. These results are in line with that already found in relation to understanding and identifying rape victims and aggressors (Osman, 2011). Research has shown that women can more easily understand and identify with victims than men (Deitz et al., 1984), and men score higher on empathy for perpetrators of aggressive acts (Jimenez and Abreu, 2003).

In this particularly articulated phenomenon, the SNSs also seem to have a role. The results made it possible to highlight that the time spent online (Park et al., 2013; Bracci et al., 2019; Parlangeli et al., 2019) is related to certain personality traits, and that stereotypes of aggressors or victims can differently emerge in online relationships. Significant differences in relation to time on social networks are evident for affective empathy and neuroticism, two factors that in connection with other variables, as the gender of the observer and the gender of the observed face, can cause the increase of judgments relative to aggressive behavior. More specifically, it appears important to emphasize that the increase in time spent interacting with social media is evidently connected with a tendency to see female faces as being more likely to have a past of aggressive behavior.

## Limitations

The study has some limitations that should be addressed.

The questionnaire was created to collect information relative to the personality traits (Big Five) and the level of empathic responsiveness (BES) of the participants in the study. However, no information was gathered regarding eventual actual experiences of participants as victims and/or aggressors; and it could be hypothesized that this could determine greater or lesser sensibility in the perception of clues of aggressiveness and victimization in the faces viewed, thus influencing the activation of the relative stereotypes.

As shown by some studies (Watkins et al., 2010), the perception of the trait of dominance (also interpreted as physical and/or social strength) in unknown faces is partially tied to the characteristics of dominance in the perceiver/observer. Less dominant men seem to be more sensitive to signs of dominance present in male faces compared to more dominant men. Correlations between the dominance of the perceiver (male) and perceived dominance in female faces do not seem to exist, however.

To discover if, and in which way, individual experience can influence the perception and elaboration of stereotypes relative to gender and perceived aggressiveness in faces with neutral expressions, this phenomenon should be the subject of subsequent and more detailed research.

Another limitation is the fact that the participants of the study were asked to evaluate the faces in reference to four variables: dimorphism: masculine-feminine, strength, past of aggression, past of victimization. In order to avoid an excessive number of questions on the questionnaire a single question was posed for each variable instead of a group of questions, which would have allowed for more in-depth examination of each of the variables considered.

The time the participants took in answering the questions regarding the evaluation of the presented faces (masculine-feminine; strong-weak, aggressor, victim) was not recorded. Analysis of the time the subjects spent to express judgments could have shed further light on the complexity of the connections between individual factors, characteristics of the stimulus, and activation or not of stereotypical associations (i.e., male—masculine—aggressor).

In trying to avoid an excessively long questionnaire there were only two questions regarding social media use (the most used and number of hours spent on social networks). Evaluating internet dependency, the level of distress caused by the impossibility to connect online, and the principal motivation for which social networks are used could have better clarified the role the Internet has on the activation of gender and victim/aggressor stereotypes.

The sample was a convenient one, and its size was not very big. A power analysis was not conducted before the study, lacking previous estimates of effect size for the dependent variables of interest (the ratings for past of aggressor/victim). The statistical methods that we used, however, tend to be more powerful than more traditional methods, and considering the relatively high number of faces seen by each participant, the current sample size should ensure a reasonable amount of power.

Lastly, it should be mentioned that the order of presentation of the 30 faces in the questionnaire was not random, but three different sequences were created, and one of these was randomly assigned to each participant. Therefore, it should be noted that, although very unlikely, in the results obtained there could be effects of the sequence of presentation of the stimuli.

## Conclusion

Exploration of the human face, even when characterized by emotionally neutral expressions, can be the root from which gender stereotypes and stereotypes relative to violent behaviors stem. Our study has confirmed the complex role—hindering or facilitating—of multiple factors involved in the elaboration of these stereotypes; factors that range from the gender of the observed face to observers’ personality traits and abilities such as empathy, extraversion, neuroticism, and openness. Moreover, it showed how intensive use of SNSs can be related to some subjective factors in facilitating the expression of both gender stereotypes and violent behavior stereotypes, as in the case of the evaluation regarding the aggressive behaviors of women, and how these judgments can be softened with a higher level of affective empathy.

Despite the limitations mentioned above, our results can have practical implications for intervention/prevention programs against offensive acts on the Internet, which could benefit from a greater awareness of the motivational function of gender stereotypes, their impact on implicit beliefs (in this case history of committed or suffered victimization), personality traits, and the role the time spent on social networks can have on this process.

## Data Availability Statement

The raw data supporting the conclusions of this article will be made available by the authors, without undue reservation.

## Ethics Statement

The studies involving human participants were reviewed and approved by the Department of Social Political and Cognitive Science of the University of Siena, which carries out the function of ethical committee in our case (September 27, 2017; report no.10/2017). Written informed consent to participate in the study was provided by the participants or where applicable, the participants legal guardian/next of kin.

## Author Contributions

MB conceived the study, analyzed relevant literature, collected the data, and wrote the introduction section. SG conducted the statistical analysis of the data, wrote the current version of the results section, contributed to writing the methods, discussion, and conclusion sections and to the revision of the other sections. EM conceived the study, analyzed relevant literature, structured the questionnaire, collected the data, and wrote the method. MM analyzed relevant literature and structured the questionnaire. PP structured the questionnaire, analyzed the data, and wrote the results and conclusion sections. OP conceived the study, analyzed relevant literature, analyzed the data, and wrote the manuscript in all its sections.

## Conflict of Interest

The authors declare that the research was conducted in the absence of any commercial or financial relationships that could be construed as a potential conflict of interest.

## Footnotes

1 https://goo.gl/forms/JvrkWkn5kXqYcZvG2
